# Editorial: Retinopathy of prematurity: an update on pathophysiology, diagnostic, methods and treatment

**DOI:** 10.3389/fmed.2026.1950313

**Published:** 2026-08-25

**Authors:** M. Elizabeth Hartnett, Shunji Kusaka, Kazuko Wada, Kazuo Itabashi

**Affiliations:** 1 Department of Ophthalmology, Stanford University, Stanford, CA, United States; 2 Department of Ophthalmology, Kindai University Faculty of Medicine, Sakai, Japan; 3 Department of Neonatal Medicine, Osaka Women's and Children's Hospital, Izumi, Japan; 4 Department of Pediatrics, Aiseikai Memorial Ibaraki Welfare Medical Center, Showa Medical University, Shinagawa, Japan

**Keywords:** retinopathy of prematurity, pathophysiology, oxygen, VEGF, regulation, angiography, multisensory stimulation, registry

Retinopathy of prematurity (ROP) is a leading cause of childhood blindness worldwide and is increasing. The appearances of ROP differ throughout the world. In areas with resources to save premature infants but with fewer resources for pre- and antenatal care and nutrition, ROP is seen in infants of older gestational ages and larger birth weights than in regions where extremely premature infants survive. Nanopremies (infants <24 weeks) have shorter gestations to develop and experience additional challenges that may influence the development of ROP. The pathophysiology and management of ROP is believed to be affected by genetic variants as well as environmental factors and resources (1).

ROP is rare and considered an orphan disease. Therefore, evidence-based studies are few because of the difficulty reaching sample sizes for analyses. In addition, evidence-based studies may be limited by combining infants with different characteristics into similar groups, potentially diluting the effects of less appreciated stimuli on outcomes or masking important factors in any one infant. Observational experience remains important in refining hypotheses. As computation of large datasets is possible with current computing, the movement toward a precision-based diagnosis becomes a potential future reality.

This issue includes ten manuscripts by experts dealing with the complex, evolving pathophysiology of ROP and its screening, diagnosis and management.

Pfeil et al. compared infants with severe ROP from Germany to those in Turkey. Both countries experienced zone II stage 3+ ROP, but Turkey had more A-ROP (aggressive ROP) than Germany. In Germany, ranibizumab was the predominant treatment, whereas in Turkey the use of laser or bevacizumab was comparable. Retreatment occurred in 30.5% after ranibizumab, 36.9% after bevacizumab and 14% after laser. Retreatment after aflibercept was reported at 44% but there were only 18 infants initially treated with aflibercept. Retreatment indications differed, with some clinicians treating for reactivated ROP and others for persistent avascular retina (PAR). Since the diagnosis of PAR is not consistent among clinicians, PAR remains a critical area for future investigations.

Huang et al. presented a Cochrane review on the refractive outcomes following treatment with anti-VEGF, laser, cryotherapy and vitrectomy. Eighty-six studies including 10,269 eyes were analyzed. The primary outcome was mean spherical equivalent and the authors included prevalence of high myopia, defined as 5.0 diopter or more, as a secondary outcome. After anti-VEGF, the pooled mean spherical equivalent was −1.9 D, after laser −3.8 D, after cryotherapy −5.8 and after vitrectomy −6.3. The authors reported 21% with high myopia after anti-VEGF, compared to 42.6% after laser. High myopia occurred in 55.4% infants after cryotherapy and 58.6% after vitrectomy. The follow up period was from 6 months to 18 years with a median of 3 years.

Optical coherence tomography (OCT) and OCT angiography were studied by Chen and Hong in 400 formerly preterm infants between 3 and 6 years of age. The spherical equivalent in eyes with severe ROP was −2.35 ± 2.75 D. The authors reported history of severe ROP, increased area of the foveal avascular zone, decreased superficial capillary density and thinner choroid in association with the development of myopia. In addition, higher serum VEGF, measured in former preterm children of 3–6 years of age, was correlated with larger FAZ area and lower superficial capillary density.

Using the Germany Retina.net ROP registry, Bründer et al. reported on 21/350 infants with aggressive posterior ROP (AP-ROP) in one or both eyes. Anti-VEGF treatment was performed at younger postmenstrual ages and required more retreatments (39%) than laser (15%). Risks for AP-ROP were low BW and GA, but not male sex or other comorbidities reported in previous studies.

Conbercept and ranibizumab were reported to have similar efficacy and reactivation rates in ROP in a study of 259 infants from China reported by Liu et al. Low postmenstrual age and zone I ROP were found to be independent risk factors associated with reactivation in this retrospective analysis. The authors concluded that infants with these characteristics require closer and longer follow up after anti-VEGF therapy with either agent.

In a preclinical study, Bailey et al. presented Cytochrome p450 (CYP46A1) expression during development and following oxygen-induced retinopathy. The rationale was based on studies that bile salts and the regulation of cholesterol products reduced experimental retinovascular pathology. CYP46A1 is involved in many pathways, including cholesterol regulation. CYP46A1 expression was developmentally regulated in the murine neonatal retina. Efavirenz, an activator of CYP46A1, reduced intravitreal neovascularization and avascular retina in the mouse oxygen-induced retinopathy model in association with elevated 24- hydroxycholesterol levels. The authors propose future studies to understand mechanisms of this pathway for vascular protection.

The regulation of oxygen is critical in preventing ROP. Smith et al. presented a cost-utility analysis of the use of oxygen blenders in Uganda in which the cost to prevent one case of ROP-driven severe visual impairment ranged from $25.55–$543.99, with a base case of

$168.92 per case averted, reflecting 5.16 DALYs averted over the 58-year average Ugandan lifespan. In comparison, prevention of malaria via mosquito net distribution was estimated in a 2020 meta-analysis to cost $50 for every DALY averted. The investigators concluded that oxygen blenders would have value in reducing vision loss from ROP.

Shen et al. conducted a comprehensive retrospective analysis of infants with GA at or below 34 weeks, divided into ROP and non-ROP groups based on fundus screening results. From clinical and laboratory testing, they reported 5 independent risks: hypertensive disorders of pregnancy, number of blood transfusions, oxygen therapy time and concentration, and blood glucose spikes in the first postnatal week. These factors were included in a nomogram estimating risk in infants born at or under 34 weeks’ gestation.

Multisensory stimulation integrates auditory, olfactory, gustatory and tactile stimuli. Tao et al. provided a comprehensive review of this method and described methods and future research needed to assess its use in ROP screening to reduce stress.

Filippi et al. provided a comprehensive discussion of the preclinical and clinical rationale for beta 3 adrenoreceptor activation to prevent ROP, adding to the body of published literature on reducing ROP by promoting physiologic vascularization, some of which is reviewed here (1, 2).

These 10 articles provide diverse thinking and studies tackling the ever-changing landscape of worldwide ROP pathophysiology and management.
